# Strategies for Controlling or Releasing the Influence Due to the Volume Expansion of Silicon inside Si−C Composite Anode for High-Performance Lithium-Ion Batteries

**DOI:** 10.3390/ma15124264

**Published:** 2022-06-16

**Authors:** Xian Zhang, Jingzheng Weng, Chengxi Ye, Mengru Liu, Chenyu Wang, Shuru Wu, Qingsong Tong, Mengqi Zhu, Feng Gao

**Affiliations:** 1 College of Chemistry and Materials Science, Fujian Normal University, Fuzhou 350007, China; qsz20201299@student.fjnu.edu.cn (X.Z.); qsz20191107@student.fjnu.edu.cn (C.Y.); qsx20190796@student.fjnu.edu.cn (M.L.); qsx20211001@student.fjnu.edu.cn (C.W.); qsx20210917@student.fjnu.edu.cn (S.W.); qstong_3503@fjnu.edu.cn (Q.T.); mqzhu@fjnu.edu.cn (M.Z.); gaofeng@fjnu.edu.cn (F.G.); 2 Fujian Provincial University Engineering Research Center of Efficient Battery Modules, Fuzhou 350007, China; 3 Fujian Provincial Key Laboratory of Polymer Materials, Fuzhou 350007, China

**Keywords:** lithium-ion batteries, silicon/carbon, anodes, volume expansion

## Abstract

Currently, silicon is considered among the foremost promising anode materials, due to its high capacity, abundant reserves, environmental friendliness, and low working potential. However, the huge volume changes in silicon anode materials can pulverize the material particles and result in the shedding of active materials and the continual rupturing of the solid electrolyte interface film, leading to a short cycle life and rapid capacity decay. Therefore, the practical application of silicon anode materials is hindered. However, carbon recombination may remedy this defect. In silicon/carbon composite anode materials, silicon provides ultra-high capacity, and carbon is used as a buffer, to relieve the volume expansion of silicon; thus, increasing the use of silicon-based anode materials. To ensure the future utilization of silicon as an anode material in lithium-ion batteries, this review considers the dampening effect on the volume expansion of silicon particles by the formation of carbon layers, cavities, and chemical bonds. Silicon-carbon composites are classified herein as coated core-shell structure, hollow core-shell structure, porous structure, and embedded structure. The above structures can adequately accommodate the Si volume expansion, buffer the mechanical stress, and ameliorate the interface/surface stability, with the potential for performance enhancement. Finally, a perspective on future studies on Si−C anodes is suggested. In the future, the rational design of high-capacity Si−C anodes for better lithium-ion batteries will narrow the gap between theoretical research and practical applications.

## 1. Introduction

As a source of economic development, energy has a significant impact on people’s quality of life. As societies expand and flourish, the demand for energy is also increasing. Due to the limited supply of conventional fuels and increasing environmental pollution, research on sustainable, green energy storage technology is ongoing [1,2,3]. For example, wind, solar, and nuclear sources have been widely developed and applied. However, as these energy resources can only provide an intermittent supply, large-scale, efficient energy storage needs to be developed to store and transmit power [4]. Currently, energy storage systems are available for various large-scale applications and are classified into four types: mechanical, chemical, electrical, and electrochemical [5,6]. Among the various energy storage technologies, electrochemical energy storage is considered one of the most promising technologies, because it is not limited by the geographical and topographical environment and can directly store and release electric energy.

Lithium-ion batteries (LIBs) have several advantages compared with other battery systems, such as aluminum ion batteries and magnesium ion batteries. First, due to the higher electronegativity of lithium vs. NHE, LIBs display higher voltage than other systems. Second, the weaker Coulombic interaction between monovalent ions accelerates kinetics. Compared with sodium-ion batteries [7] and potassium-ion batteries [8], LIBs are studied by many researchers, due to their high energy density, long cycle life, and sustainability. Concerning the maturity and feasibility of the current technologies, LIBs are one of the most attractive solutions in the field of electronic terminal equipment, due to the advantages of relatively high gravimetric and volumetric energy densities [9], high power density [10], long cycle life, light weight, good rate capability, low self-discharge rate, high working voltage, and safety [11]. Advancements in research have introduced a series of new methods (e.g., solid-state approach [12], 3D printing [13], etc.). Whereby, 3D printing is an additive manufacturing (AM) technique for the development of advanced materials, architectures, and systems for a broad range of applications: energy [14], electronics [15], and engineered composites [16,17]; it promises the maturity of LIBs technology and high utilization in the market [18,19].

LIBs contain four parts: a cathode, an anode, an electrolyte, and a separator. The anode plays a significant role in the reversible storage of Li^+^ [20,21]. In an effort to boost the electrochemical performance of LIBs and expand their application, researchers have focused on developing superior anode materials for LIBs, to increase their cycle life [22,23,24].

Silicon is considered among the foremost anode materials with potential for next-generation LIBs [11]. However, during cycling, the volume of the silicon anode material expands (>300%) [25,26], the silicon particles crack and disintegrate, and then the inner resistance of the LIBs increases, diminishing its capacity [27,28,29,30].

Carbon material has a relatively stable structure, limited volume expansion (<10%) upon cycling [31,32], and excellent electrical conductivity, mechanical flexibility, and thermal and chemical stability [33,34]. It is compatible with silicon: the additional carbon layer not only acts as a buffering shell to mitigate volume changes, but it also decreases the contact resistance between Si particles with the current collector [35,36]. In addition, Si/C coupling combines the high specific capacity of Si and the high conductivity of carbon, while reducing the volume changes of Si during cycles to ensure the structural integrity of the electrode [37].

Various types of anode materials for LIBs and the volume expansion fade mechanism of the Li-Si system are provided in Section 2. In Section 3, different structural innovations are described, such as coated core-shell structure, hollow core-shell structure, porous structure. For each structure, the formation of carbon layers, cavities, and chemical bonds are highlighted. And some Si−C composite mitigation mechanisms on the volume expansion of silicon particles are discussed. Some future perspectives on practical Si/C anodes are briefly summarized Section 4.

## 2. Anodes Materials for LIBs

### 2.1. Graphite-Type Anode Materials

Graphite-type anode materials can form LiC_6_ intercalation compound and have a stable electrochemical performance [38,39], but they fail to cater to the ever-growing demands for high specific capacity and high rate capability; this can be attributed to their limited theoretical capacity of 372 mAh g^−1^ in LIBs [40,41,42].

### 2.2. TMOs

Consequently, transition metal oxides (TMOs) have been developed as alternative anode materials for LIBs, due to their good corrosion resistance, chemical stability, and attractive theoretical specific capacities (up to 1000 mAh g^−1^, which is 2–3 times higher than that of commercial graphite anodes), induced by a conversion-type reaction [43,44]. However, conventional TMO anodes usually suffer from low electrical conductivity and large volume changes during electrochemical cycling, which can result in electrode pulverization and, therefore, rapid capacity decay [45].

### 2.3. Phosphorus (P)-Anode Materials

Hence, scientists have devoted significant efforts toward designing new materials or novel structures with existing materials, and which have large earth abundance and exhibit favorable electrochemical properties, such as silicon (Si)- and phosphorus (P)-anode materials. The storage mechanism of metal phosphides for lithium ions includes three types (Equations (1)–(3)), i.e., intercalation, conversion, and alloying:

Intercalation reaction: M_x_P_y_ + zLi^+^ + ze^−^ ⇋ Li_z_M_x_P_y_ (1)

The characteristic of the intercalation mechanism is that the insertion of Li^+^ does not significantly damage the structure of M_x_P_y_ and, thus, maintains its cycle performance. Nevertheless, the specific capacity is relatively low [46,47].

Conversion reaction: M_x_P_y_ + 3yLi^+^ + 3ye^−^ ⇋ yLi_3_P + xM (2)

For M_x_P_y_ relying on the conversion mechanism, phosphorus is converted into Li_3_P, and the metal element, M, is dispersed in the form of nanoparticles in the Li_3_P matrix. Although the insertion of Li^+^ will destroy the M_x_P_y_ structure in this case, the presence of M can help buffer the structural change and improve the conductivity of composites [48,49].

Alloying reaction: M_x_P_y_ + (3y + bx)Li^+^ + (3y + bx)e^−^ ⇋ yLi_3_P + xLi_b_M (3)

A trait of M_x_P_y_ in which the alloy mechanism dominates is that, in addition to the conversion of phosphorus into Li_3_P, M is also alloyed to produce Li_b_M. The b value can be obtained from the phase diagram of Li-M binary alloy. Although the insertion of Li^+^ will completely destroy the structure of M_x_P_y_, these materials have the highest capacity among all metal phosphides [50]. However, when it comes to practical applications, phosphide anodes generally suffer from the following issues: rapid capacity decay, short cycling life, low initial Coulombic efficiency (ICE), and high preparation cost; while the low ignition point of phosphorus has certain dangers.

### 2.4. Silicon (Si) Anode Materials

Silicon as an anode material has the following benefits:

(1) Highest theoretical specific capacity (approximately 4200 mAh g^−^^1^), which is much higher than other types of anode materials [51,52];
(2) Abundant reserves (the second-highest content in the Earth’s crust), affordable, environmentally friendly, and non-toxic [53];
(3) Discharge potential is low (<0.5 V) [54,55], which mitigates safety issues, due to the growth of lithium dendrites [56,57,58].

However, during expansion, significant stress is generated in the battery interior, which causes the pole piece to extrude. As the cycle progresses, the pole piece can easily fracture [59,60]. This stress may also reduce the internal cavity of the battery and limit the movement of Li^+^, causing the metallic lithium to precipitate, which can be dangerous [40]. As the volume expands, the solid electrolyte interface (SEI) membrane on the surface of the silicon anode will rupture, forcing the silicon material to reform its SEI while charging and discharging. Repeated ruptures and formations accelerate the consumption of Li^+^ and electrolytes and, ultimately, lead to an increase in the internal resistance and a rapid decrease in capacity [61,62,63]. Moreover, due to the poor conductivity of silicon, the release of capacity will be limited at a high rate. These reasons restrict the further application of silicon as an anode material. For this reason, solving the challenges of volume expansion and poor electrical conductivity in silicon has become a research hotspot, with a particular interest in the use of silicon-carbon composite materials.

### 2.5. Fading Mechanism of Volume Expansion on Performance

Studies have shown that the electrochemical reaction of silicon involves a lithiation-delithiation process. With the gradual intercalation of lithium, the alloy phases (e.g., Li_12_Si_7_, Li_7_Si_3_, Li_13_Si_4_, Li_22_Si_5_, etc.) are formed in a sequence [64,65,66]. The theoretical specific capacity (4200 mAh g^−1^) of Si is estimated by the composition of the alloy phase with the highest lithium content [67]. The alloying-dealloying reaction of silicon may be summarized as in Equation (4):

Si + xLi^+^ + xe^−^ ⇋ Li_x_Si  (0 ≤ x ≤ 4.4) (4)

**Figure 1:**
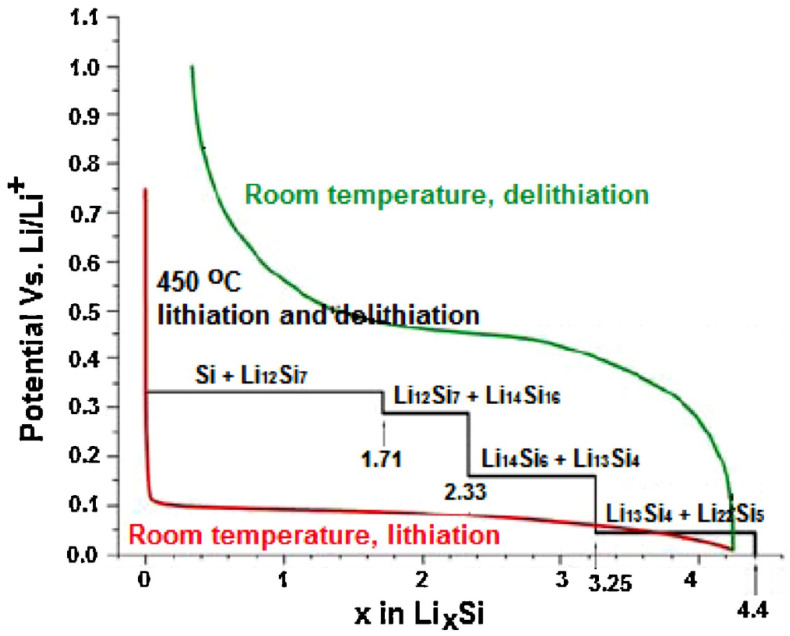
Si lithiation/delithiation curves at room temperature and 450 °C [69]. Reprinted with permission from Ref. [69]. Copyright 2012, Nano Today.

During the process of Li^+^ insertion, Li^+^ passes through the diaphragm to the surface of the Si particles, to make an Li-Si alloy, Li_x_Si. This process can effectively avoid the co-insertion of the electrolyte and stabilize the uniformity of the electrolyte and the structure of the electrode. As shown in Figure 1, from the voltage-capacity curve of the silicon electrode material, the voltage drops rapidly to 0.2 V during discharge, and a longer Li^+^ insertion platform appears. During the process of Li^+^ extraction, a dealloying reaction occurs, and the Li^+^ extraction potential is in the range of 0.4−0.5 V. As compared to carbon materials, the Li^+^ insertion/extraction potential is slightly higher than that of carbon materials, and this prevents the precipitation of lithium dendrites as anode materials, so it is safer with a narrower voltage range. Therefore, it is appropriate as an anode material for LIBs [68].

**Figure 2:**
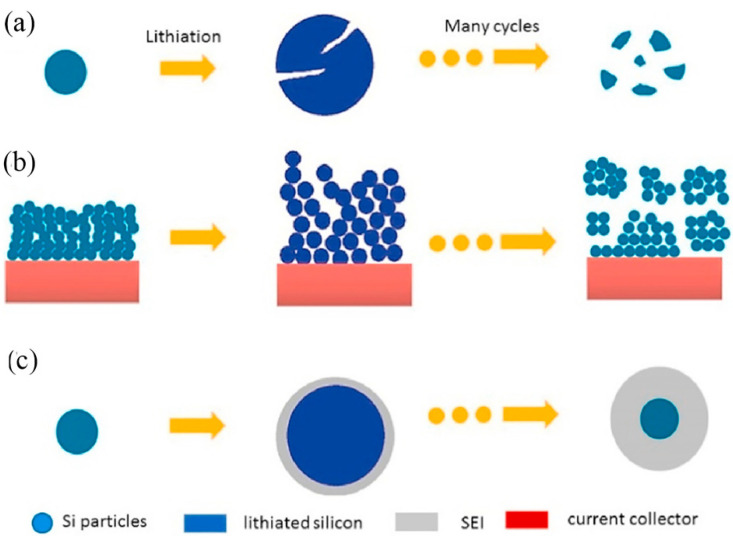
Various failure mechanisms of Si-based electrodes: (**a**) particle pulverization; (**b**) the repeated generation and destruction of SEI; (**c**) collapse of the electrode structure [76]. Reprinted with permission from Ref. [76]. Copyright 2021, J. Power Sources.

Ideally, if the amount of inserted and extracted Li^+^ is equal each time, indicating that the process is fully reversible, then a good capacity retention is exhibited, in terms of electrochemical performance. This state is based on the ability to limit the depth of Li^+^ insertion, which is not conducive to the release of specific capacity [70]. During the process of alloying, the crystal silicon is gradually transformed into an Li-Si alloy, and the Si-Si covalent bond in the crystal is gradually replaced by an Li-Si bond, and finally the Li1_5_Si_4_ alloy phase is completed. During the process of dealloying, the Li-Si bond becomes an Si-Si covalent bond again. With the continuous process of alloying and dealloying, the bond length of the Li-Si bond becomes much larger than that of the Si-Si covalent bond, which results in a significant change in volume. The expansion of Li-Si alloy can be accommodated by relatively soft and ductile carbon materials [71]. The volume expansion of the silicon anode material causes three problems, as illustrated in Figure 2:

(1) Particle pulverization: During the process of alloying and dealloying, the repeated phase transition between crystal silicon and Li-Si alloy produces significant stress. After several cycles, when this stress reaches the threshold of the silicon material, the silicon particles are pulverized and can no longer store lithium (Figure 2a).
(2) Repeated rupture and formation of the SEI: SEI prevents the contact between the active material and the electrolyte; without this interface, the two will react and consume each other. Since the volume expansion of Si can rupture the SEI, and thus expose the active material to the electrolyte, a large number of active materials and electrolytes are consumed until a new SEI is formed, resulting in a sharp reduction in the capacity of the LIBs over time (Figure 2b) [72,73]. In addition, the recurrent formation and destruction of the SEI layer can further the formation and growth of Li dendrites [74,75].
(3) Silicon electrode structure changes: Each charging-discharging cycle causes a phase transformation between the crystalline silicon and the Li-Si alloy. This repetitive expansion and contraction of the silicon particles changes the volume of the whole electrode over time. Eventually, the silicon particles fall off in the current collector and cannot be alloyed, leading to a rapid decay of capacity and cycle performance (Figure 2c).

## 3. Mitigation of Volume Expansion

**Figure 3:**
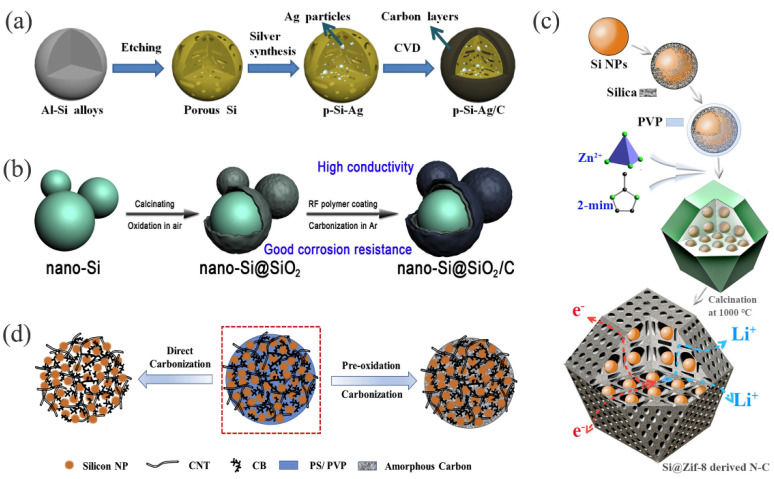
Schematic diagram of the different structures of Si–C composites: (**a**) coated core-shell structure [77]. Reprinted with permission from Ref. [77]. Copyright 2018, J. Power Sources. (**b**) hollow core-shell structure [78]. Reprinted with permission from Ref. [78]. Copyright 2018, J. Alloys Compd. (**c**) porous structure [79]. Reprinted with permission from Ref. [79]. Copyright 2020, Appl. Surf. Sci. (**d**) embedded structure [80]. Reprinted with permission from Ref. [80]. Copyright 2018, Carbon.

Various carbon materials have been developed to address the adverse reactions caused by the volume expansion of silicon. An Si/C anode combines the high capacity of silicon and the high conductivity of carbon and, therefore, alleviates the volume change of silicon during cycling and ensures the integrity of the structure. As shown in Figure 3, based on the structure of a Si−C composite anode material, it can be divided into four aspects: the coated core-shell, the hollow core-shell, the porous structure, and the embedded structure.

### 3.1. Coated Core-Shell Structure

Coated core-shell silicon-carbon composites use silicon particles as the core and uniformly coat the outer surface of the core with a carbon layer. The carbon layer not only allows an electrical contact between Si particles and buffers the volume expansion of Si, but it also conjointly reduces the contact between the Si surface and the electrolyte; decreasing the decomposition of the electrolyte and improving the cycle life of the electrode (Table 1). Initially, there were few options for Si−C composites; typical methods included mixing Si powder with various carbonaceous materials, to obtain coated Si−C composites by ball milling [28,81]. An Si−C composite material with a coated core-shell structure has the following advantages: (1) improves the electrical conduction of electrons and ions; (2) provides mechanical support to adapt to the volume expansion of Si; and (3) isolates the Si and the electrolyte, to form a stable SEI film, thereby improving the first Coulombic efficiency (CE) [82].

#### 3.1.1. Inorganic Carbon Source

Coated Si−C composites are obtained by depositing different carbon materials (e.g., graphite [83], graphene [84], carbon nanotube [85], etc.) on the surface of silicon nanoparticles. Due to the limited volume expansion, the carbon layer mitigates silicon’s volume expansion and reduces the stress, which improves the structural stability and stabilizes the formation of SEI. This effectively improves the electrochemical performance and battery life.

**Figure 4:**
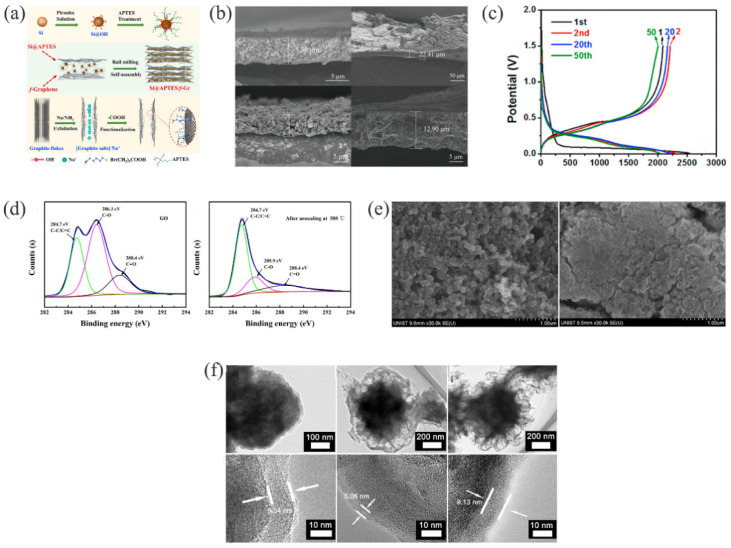
(**a**) Schematic diagram of the synthesis of Si@APTES/f−Gr composite [92]. Reprinted with permission from Ref. [92]. Copyright 2018, Carbon. (**b**) Cross-sectional SEM images of Si and Si@MnO_2_@rGO, before and after 150 cycles [99]. Reprinted with permission from Ref. [99]. Copyright 2022, Nanomaterials. (**c**) mSi−C composite charge-discharge voltage profiles [109]. Reprinted with permission from Ref. [109]. Copyright 2016, J. Power Sources. (**d**) XPS analysis of GO and RGO [98]. Reprinted with permission from Ref. [98]. Copyright 2018, Powder Technol. (**e**) SEM images of mSi−C composite, before cycling and after 100 cycles [109]. Reprinted with permission from Ref. [109]. Copyright 2018, J. Power Sources. (**f**) TEM images of the CoMOF−D@Si@C electrode in a fully charged state at first cycle, after 50 cycles, after 100 cycles [115]. Reprinted with permission from Ref. [115]. Copyright 2021, Electrochim. Acta.

Graphene, which was first isolated in 2004 [86], is a monatomic thin layer of carbon atoms with sp^2^-bonds [87]. It is an ideal nanostructure matrix for carrying lithium, due to having a large specific surface area, good flexibility and mechanical strength, excellent electrical conductivity, and chemical stability [88]. In silicon-based anode materials, it adapts to the volume changes and stress during the alloying reaction, and it is a good buffering material to alleviate volume expansion [89]. For example, the surface of silicon nanoparticles coated with crumpled graphene did not fracture due to the volume expansion/contraction during the charging/discharging process [90]. DMSiG [91] remained intact after 100 cycles, indicating that its structure was strong enough to withstand volume changes. Si@APTES/f−Gr [92] composite materials formed stable covalent bonds between silicon nanoparticles and graphene, via the introduction of amino and carboxyl groups (Figure 4a). The stability of the structure was ensured, and this mitigated the volume changes. The (Si@Si_3_N_4_)/GrNCs [93] electrode had a high capacity of 827 mAh g^−1^ after 450 cycles at the ultra-high current density of 2 A g^−1^. The results showed that this unique structure not only provided space and flexibility to buffer the volume changes of Si during lithiation/delithiation [94], but it also played a key role in improving the conductivity and electrical connection, as well as in promoting the rapid transport of Li^+^ and charge storage in the electrode [93]. The electrode had a good cycle stability and rate performance.

Graphene oxide (GO) is a kind of graphene, and it has oxygen-containing functional groups to maintain the activity of its properties and can change its properties by reacting with other oxygen-containing functional groups. After a series of chemical reactions, reduced graphene oxide (RGO) is obtained by removing its oxygen-containing functional groups. The structure of this carbon material is relatively stable [95,96]. As shown in Figure 4d, X-ray photoelectron spectroscopy (XPS) analysis showed that after annealing at 500 °C, the peak intensity of C-O and C=O (286.3 and 288.4 eV, respectively) had been significantly weakened, indicating that GO had successfully transformed into RGO [97]. Su et al. prepared multi-layer carbon-coated silicon Si@C@RGO [98]. Liu et al. successfully prepared an Si@MnO_2_@rGO anode using a hydrothermal method [99]. As shown in Figure 4b, after 150 cycles, the volume expansion of the Si@MnO_2_@rGO electrode was only 59%, while the bare silicon electrode was as high as 198%. A coating of rGO not only suppresses the volume expansion of the electrode, but it also prevents the agglomeration of internal materials and improves the conductivity of the electrode, to accelerate the transport of Li^+^ [100]. Nitrogen doping in an Si@N-doped rGO/CNF [101] anode material further improved the conductivity of rGO and improved the gap between rGO and silicon nanoparticles. It showed a better buffering effect for silicon volume expansion and significantly improved the long-cycle performance of the electrode material [102].

Carbon nanotubes have shown a unique, three-dimensional conductive morphology and have sufficient mechanical strength to penetrate the interior of the active material and provide excellent conductivity [103]. Silicon nanoparticles, via chemical vapor deposition (CVD), were deposited on the surface of carbon nanotubes (CNTs) vertically arranged on a stainless steel substrate [104]. The formation of covalent bonds between the CNTs and the silicon nanoparticles connected the carbon nanotubes to the silicon, which not only alleviated fracturing during delithiation, but also increased the electron transport to the active material [105]. However, the bonding between the Si and the CNTs was weak. As a result, the silicon nanoparticles were easily detached from the surface of the carbon nanotubes, resulting in rapid capacity decay [106]. To stabilize the silicon nanoparticles on the surface of carbon nanotubes, sandwich CNTs/Si−C composites were obtained, by coating a layer of carbon on the surface of the CNTs/Si using a hydrothermal method [107]. The inner CNTs provided mechanical support for a stable structure, and the outer carbon layer promoted the stable formation of the SEI. The combined effect not only alleviated the volume expansion during cycling, but it also improved the electrical conductivity of the silicon nanotubes. A CNT–Graphene–Si [108] composite combined the advantages of carbon nanotubes and graphene. This unique structure had a large number of gaps, which provided enough space for the volume expansion of the silicon and promoted the transmission of electrons.

A silicon-carbon composite (MSI−C) with an agglomerate structure was prepared by coating a layer of conductive carbon on the surface of nano-silicon [109]. The first charge capacity of the charge-discharge platform showed that most of the Si particles (approximately 82.3%) were involved in the charge-discharge process, and the voltage nearly overlapped during the 20th and 50th cycles (Figure 4c). The results indicated that this porous structure buffered the volume expansion of silicon [110]. As shown in Figure 4e, the scanning electron microscope (SEM) images of mSi−C composites captured before and after cycling were still mostly unchanged. This, once again, illustrated the uniqueness of this kind of structure [111]. Metalorganic frameworks (MOFs) have a larger specific surface area, tunable pore structures, and enhance the connection between host and guest materials [112]. They have been widely used for the anodes in LIBs [113,114]. Yan et al. successfully synthesized a core-shell material (CoMOF−D@Si@C) by uniformly depositing silicon on the surfaces of pyrolytic MOFs and then coated them with a carbon shell [115]. The transmission electron microscope (TEM) analysis in Figure 4f shows that the volume expansion of the first cycle was slightly larger (9.54 nm), in order to adapt to the structural change, but the expansion degree after 50 cycles was greatly reduced, at only 53% of the first cycle. There was a gap between the outer carbon shell and the inner silicon, which ensured that the carbon shell would not crack during expansion [116]. After 100 cycles, although a small amount of carbon shell had cracked and slightly increased the thickness of the SEI, the overall structure was not damaged. The electrode material had a capacity of 684 mAh g^−1^ after 1200 cycles, even at a high current density of 4 A g^−1^, due to the effective reduction in silicon volume expansion and the improvement in electrical conductivity.

#### 3.1.2. Derived Carbon Source

Derived carbon is obtained by carbonizing various organic compounds (e.g., sucrose [117], glucose [118], polyacrylonitrile or polystyrene [119], polyvinylidene fluoride (PVDF) [120], polydopamine [116,121], and resorcinol-formaldehyde resin [122], etc.), to achieve similar effects with inorganic carbon sources. This provides another method for the diversified preparation of silicon-carbon composites that can then be coated with a single or a double layer of carbon.

This type of structure provides protective and performance-enhancing benefits for silicon-based electrodes [123]. The carbon layer, not only prevents the agglomeration of silicon particles and limits contact with the electrolyte, but it also enhances the electrical conductivity of the structure. However, when placed under stress, due to silicon’s volume expansion during cycling, the carbon shell ruptured, leading to the repeated formation of SEI and, thus, a rapid decay of CE and capacity [124,125,126]. For example, for Si@C composites synthesized with p-phenylenediamine [127] and RF (resorcinol-formaldehyde) [128] as carbon sources, the capacity retention rates were 68.2% after 100 cycles under 100 mA g^−^^1^ and 29.8% after 10 cycles under 50 mA g^−1^, respectively. The rupture of the carbon shell after cycling was the cause of the reducing capacity [128]. The results showed that the buffering effect of a single carbon coating on the volume expansion of silicon was limited, resulting in insufficient structural stability of silicon-based electrodes for a long cycle life.

To prepare a silicon-based electrode with a long cycle life, a double-layer coating of silicon further improved the suppression of the volume expansion. For example, Si/C−G [129] used hydrogen-bonding to create a carbon layer, and graphene provided a double buffer for the volume change during alloying. Under the combined action of the SiO_2_ and C layers, Si@C@SiO_2_ [130] reduced the degree of lithiation and the stress of the volume expansion [120]. Liu et al. successfully synthesized a G@Si/SiO_2_ NPs/C [131] hybrid composite by introducing an active additive, SiO_2_. The introduction of SiO_2_ resulted in the electrode generating inert components (Li_2_O and Li_4_SiO_4_) during lithiation. The inner graphite and outer carbon layers formed a 3D network to buffer the silicon and SiO_2_, reducing the agglomeration and volume changes between particles and protecting the structure during long-term cycling. After 200 cycles, the thickness of the electrode had increased by 46%. Therefore, the G@Si/SiO_2_ NPs/C electrode had a higher tolerance for volume expansion.

**Figure 5:**
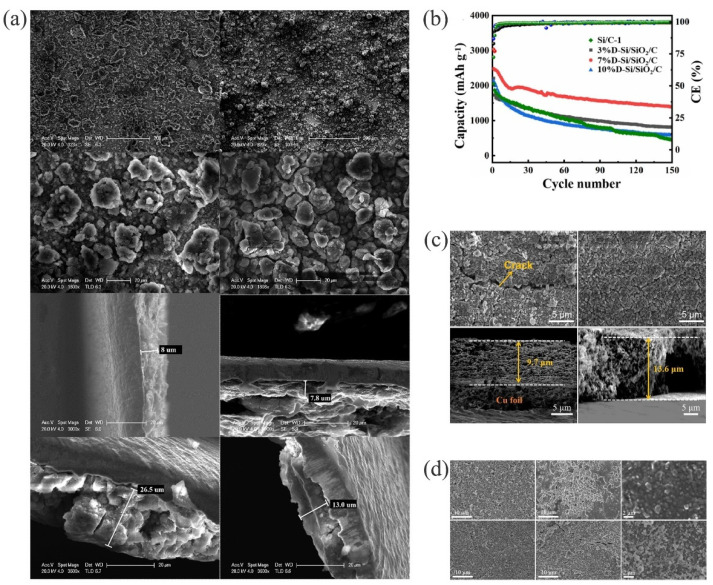
(**a**) SEM images of Si@C/rGO and Si@C/(15)Sn@C/rGO after 60 cycles and SEM images of cross-sections of Si@C/rGO and Si@C/(15)Sn@C/rGO, before and after 60 cycles [143]. Reprinted with permission from Ref. [143]. Copyright 2021, J. Mater. Sci. Technol. (**b**) Cycling performance of Si/C−1, 3% D−Si/SiO_2_/C, 7% D−Si/SiO_2_/C, and 10% D−Si/SiO_2_/C anode at a current density of 200 mA g^−1^ [147]. Reprinted with permission from Ref. [147]. Copyright 2021, Electrochim. Acta. (**c**) SEM images of pristine Si and 7%D−Si/SiO_2_/C at after 35 cycles, the cross-sectional SEM images of the 7% D−Si/SiO_2_/C electrode, before and after 35 cycles [147]. Reprinted with permission from Ref. [147]. Copyright 2021, Electrochim. Acta. (**d**) SEM images of Si@C@ZIF−67−800N and Si@C, before and after 100 cycles [151]. Reprinted with permission from Ref. [151]. Copyright 2019, Energy Storage Mater.

The volume expansion of the silicon electrode was further alleviated by doping with metal particles, such as Ni [132], Cu [133,134,135], Ag [136], Sn [137], etc. In particular, Sn has similar properties to silicon, but a better conductivity and ductility. As compared to Si/Sn [138] composites, carbon materials separated Si from Sn, which provided a more stable cycle performed with the Si−Sn−C [139]. The existence of carbon not only increased the capacity but also provided a conduction pathway and structural mechanical support [140,141,142]. Zhu et al. successfully prepared a 3D conductive structure of Si@C/Sn@C/rGO using reduced graphene oxide as an anchor [143]. The large-size Sn and carbon shell worked together, significantly alleviating the volume expansion and agglomeration of the small-size silicon. When comparing the electrode changes of Si@C/rGO and Si@C/(15)Sn@C/rGO anodes before and after 60 cycles, as shown in Figure 5a, the volume expansion of Si@C/rGO anode was significant. The degree of expansion was as high as 331.25%. The Si@C/(15)Sn@C/rGO anode was 166.67%, and there was no obvious agglomeration. In addition, it had an ultra-high capacity, nearly 1000 mAh g^−1^, after 300 cycles at a current density of 1 A g^−1^.

In particular, the surface activity and electrochemical performance of Si/C anodes were enhanced by heteroatom doping. Since the atomic radius of nitrogen is similar to that of carbon atoms, it easily replaces carbon atoms when preparing nitrogen-doped carbon materials; therefore, it has been the most studied among all atom types [144,145]. Li et al. obtained Si@N−C composites with a core-shell structure using the carbonized precursor Si@PANI [146]. The nitrogen-carbon layer not only improved the conductivity, but it also prevented the reaction between silicon and the electrolyte, while maintaining the structural stability and reducing silicon’s volume changes. Other doping atoms were tested for their performance in LIBs. Chen et al. successfully prepared P-Doped Si/SiO_2_/C using polyethanol butyral (PVB) as a carbon source and phosphoric acid as a dopant [147]. The carbon layer produced by the PVB decomposition not only suppressed the volume expansion of silicon, but it also increased the surface conductivity. The introduction of phosphoric acid as a dopant not only improved the intrinsic conductivity of the material [148,149], but it also promoted the formation of the SiO_2_ layer on the surface of the silicon, while eliminating the SiO_x_ with a low degree of oxidation and reacting with Li^+^ to generate Li_2_Si_2_O_5_, to reduce the volume expansion of silicon [150]. However, changes to the amount of phosphorus doping had a significant impact on capacity. Figure 5b shows the cyclic performance of a D−Si/SiO_2_/C electrode doped with 3%, 7%, and 10% phosphoric acid. The 7% D−Si/SiO_2_/C had the best cycling performance, as the proper amount of phosphorus-doping resulted in a better intrinsic conductivity for the electrode. However, excessive doping with phosphoric acid led to the formation of various non-conductive substances (e.g., P_2_O_5_, metaphosphate and pyrophosphate, etc.) on the surface of the electrode; thus, affecting the cycle performance of the electrode. After 35 cycles, the structure of the electrode doped with phosphorus had not significantly changed, and the degree of expansion was 40%. These results indicated that the 7% D−Si/SiO_2_/C electrode had a good cycling stability (Figure 5c).

Liu et al. successfully synthesized a double-carbon-coated multi-core mesoporous composite Si@C@ZIF−67−800N [151] by co-doping with cobalt/ nitrogen. The introduction of mesoporous increased the transmission speed of ions/electrons and provided sufficient space for the storage of lithium ions. Cobalt and nitrogen co-doping provided carriers for the electrons, catalyzed the carbonization of ZIF−67, and effectively prevented the agglomeration of Si@C particles. There was a gap between the core particles of Si@C and the derived carbon shell of ZIF−67, and the volume expansion of silicon was affected by the interaction with the double carbon coating. The thickness of the Si@C and Si@C@ZIF−67−800N electrodes before cycling was 10.6 μm and 13.7 μm, respectively, and the volume expansion rate after 100 cycles was 56% and 27%, respectively. Figure 5d shows that the structure of Si@C@ZIF−67−800N and Si@C remained intact before cycling, and the former had a larger granularity. After 100 cycles, the Si@C electrode had large cracks, and its size expansion was more obvious. In contrast, the Si@C@ZIF−67−800N electrode remained intact and had significantly less volume expansion. As compared to the single-layer carbon-coated Si, the double-layer carbon-coated Si had a higher capacity and better cycling stability [152].

**Table 1:** Electrochemical performance of coated core-shell silicon/carbon anodes for LIBs.

Anode Material	Preparation Method	Current Density ^1–2–3^ (mA g^−1^)	Initial Discharge Capacity (mAh g^−1^)	Cycle Number	Cycling Performance/Capacity Retention (mAh g^−1^/%)	Rate Capability (mAh g^−1^)	Refs.
Si@crumpled graphene	Modified Hummers method	N/A–1000–200		940	940	1200	[90]
DMSiG	Sponge template method, Magnesium reduction method	100–500–2000	2969	250	1318	910	[91]
Si@C@RGO	Two-step in situ carbon coating	100	1474.9	40	722.1	1498.8	[98]
CNTs/Si/C	Hydrothermal method	500	1280.4	1000	1508.5	1287	[107]
CNT−Graphene−Si	Modified Hummers method	100–1000–100	2155	500	1160, 88.5%	1800	[108]
mSi−C	Aerosol process, Carbon layer deposition	400–400 –12,000	2084	100	1694	906	[109]
CoMOF−D@Si@C	CVD	400–4000–10,000	1966	1200	648	957	[115]
Si/C−G	Modified Hummers method	375.9–375.9–17,895	1910.5	700	1196.1, 65%	507.2	[129]
Si@C@SiO_2_	Wet chemical process	200	880	305	89.4%	756	[130]
G@Si/SiO_2_ NPs/C	One-pot co-sol–gel process	100–500–100	548	800	607, 92%	815	[131]
Si@C/Sn@C/rGO	Solution impregnation, Hydrogenation reduction	500–1000–1000	2680	300	1000	1792	[143]
P-Doped Si/SiO_2_/C	Solid state method	200–200–100	2860.3	150	1389.8	2261.8	[147]
Si@C@ZIF−67−800N	Sol-gel, MOF self-template method, High temperature pyrolysis	200–1000–1000	1918	300	852	1253	[151]
Si@C	Hydrothermal method	420–2100–8400	2956	800	1127	668	[153]
Si/PDAC/rGo	Organic pre-coating method, High-temperature pyrolysis	100–1000–2000	1948	43	828, 78.7%	717	[154]
Si@SiO_x_/C	Ball-milling, Post annealing	210	1777	300	783		[155]

Current density ^1–2–3^: ^1^ indicates that the first value is the current density of the first discharge capacity; ^2^ indicates that the second value is the current density of the cycle performance; ^3^ indicates that the third value is the current density of the rate capacity. If there is only one value, this means that the current densities of the three are the same.

As mentioned above, a coating of carbon material mitigates the agglomeration of silicon particles and the volume expansion of the material during lithium insertion/extraction, which improves the overall performance of the anode material. However, this assumes that the carbon-coated surface of the silicon is smooth and intact. Volume changes can cause the core-shell particles to expand and crack the surface carbon layer, resulting in the collapse of the composite structure and a rapid decrease in cyclic stability. Therefore, factors that affect the performance of the composite materials should be considered in the design of such structures: (1) whether the particles are uniformly dispersed in the inner layer; (2) the thickness of the carbon shell; (3) the choice of carbon source; and (4) the structural design of the outer layer [81]. To overcome any issues, a core-shell structure with cavities has been studied under various conditions.

### 3.2. Hollow Core-Shell Structure

The hollow core-shell structure is a multiphase nano-composite formed by introducing a cavity between the silicon core and the carbon shell through technical means and according to the design of the coated core-shell structure [116]. Hollow core-shell silicon–carbon composites have a unique Si@void@C shell configuration that, not only has the advantages of an ordinary core-shell structure, but its cavity can also adapt to the volume expansion of silicon. This ensures the stability of the overall structure during the charging–discharging process (Table 2). The external carbon shell does not have direct contact with the electrolyte and the internal silicon nanoparticles, which allows it to effectively evade any side reactions and results in a more stable, durable SEI [156]. Such structures are typically prepared using a template, and the steps include the following: (1) the preparation of the template; (2) the deposition of carbon onto the template; and (3) the removal of the template.

#### 3.2.1. SiO_2_ Template Type

**Figure 6:**
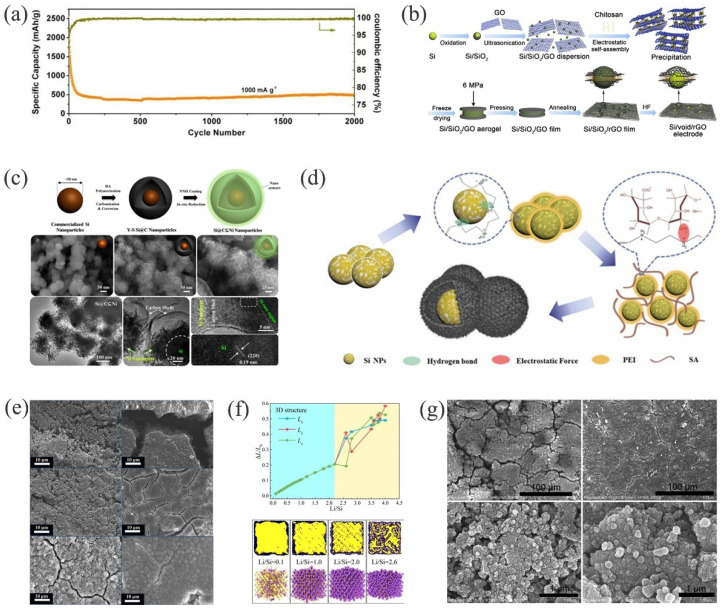
(**a**) Long-term cycling performance of the Si@HC−1 [159]. Reprinted with permission from Ref. [159]. Copyright 2020, J. Alloys Compd. (**b**) Schematic diagram of synthesis of Si/void/rGO electrode [160]. Reprinted with permission from Ref. [160]. Copyright 2021, Materials. (**c**) The synthesis process of Si@C ⊆ Ni, SEM images at different stages and TEM structure analysis [162]. Reprinted with permission from Ref. [162]. Copyright 2018, ACS Appl. Mater. Interfaces. (**d**) Preparation process of Si@void@C [175]. Reprinted with permission from Ref. [175]. Copyright 2018, Chem. Eng. J. (**e**) SEM images of the surfaces of the Si NPs, Si@C, and Si@void@C electrodes after 5 and 200 battery cycles [175]. Reprinted with permission from Ref. [175]. Copyright 2018, Chem. Eng. J. (**f**) The MD simulation results of Si@C−Ni [75]. Reprinted with permission from Ref. [75]. Copyright 2021, Energy Storage Mater. (**g**) SEM images of the n-Si and Si@C electrodes after 50 cycles [191]. Reprinted with permission from Ref. [191]. Copyright 2021, J. Alloys Compd.

The preparation of the hollow core-shell structure generally adopts the sacrificial SiO_2_ template method, and the cavity is obtained by acid-base etching [157,158]. Using Si NPs and Si@C nanoparticles as the core and SiO_2_ as the sacrificial layer, the Si@void@C [116] and Si@C@void@C [158] are obtained. The cavity between the carbon shell and the silicon core of this type of structure not only provides enough space for the expansion of the silicon but also ensures the stability of the carbon shell, so as not to destroy the surface SEI. For the Si@C@void@C structure, the extra carbon shell inside, not only increases the conductivity of the electrode, but also effectively protects the Si core from corrosion when etching the SiO_2_ to create cavities. The empty space between the Si core and the carbon shell serves as a buffer for volume changes. To better adapt to the volume expansion of Si, it is important to control the size of the space. Zhang et al. successfully synthesized controllable Si@HC [159] composites by a simple one-pot-synthesis method. By adjusting the size of the void space and the thickness of the carbon shell, they found that when the thickness of the carbon shell was 12 nm and the width of the empty space was 60 nm (Si@HC−1), the electrochemical performance of the electrode was optimal. A capacity of 500 mAh g^−1^ was achieved after 2000 cycles at an ultra-high current density of 1000 mA g^−1^ (Figure 6a). As shown in Figure 6b, Shao et al. successfully synthesized an Si/void/rGO [160] anode material with a self-supporting function by using electrostatic interaction. The space surrounding the Si NPs sufficiently buffered the volume changes during the cycles and reduced the electrode damage caused by the volume expansion of Si. At the same time, due to the good flexibility and mechanical properties of graphene, it played a similar role in regulating the size of the space during the Si volume changes. The reduced graphene oxide had an excellent electrical conductivity and was cross-linked to form a conductive network that greatly improved the overall electrical conductivity of the composite material.

However, due to the carbon-shell fatigue and damage during repeated charging and discharging, the above problems have not yet been fully resolved [161]. Due to the excellent toughness and ductility of Ni, Jiang et al. combined it with Y−S Si@C nanoparticles to obtain an armored Si@C ⊆ Ni anode material [162]. Figure 6c shows the synthesis process of the Si@C ⊆ Ni structure. The Si@C mixture was uniformly embedded in the interconnected Ni nano-framework, and the silicon nano-particles were well coated in the carbon and Ni layers. The Ni and C layers were continuous and complete, and their integration inhibited the expansion and aggregation of the silicon. The single-layer Ni had the advantages of minimal thickness, good air permeability, and lighter weight, which accelerated the transfer of electrons, while ensuring a high gravimetric energy density in LIBs [163,164,165]. Sun et al. used Si@void@mesoporous silica nanostructures as templates to prepare Si@DC with a double-layered cavity structure [118]. The cavity in the double shell was sufficient for the expansion of silicon. The outer carbon shell acted as the SEI on the surface of the stable structure and protected the inner shell and silicon core from the external conditions [122]. The ultra-high surface area not only provided sufficient active sites for lithium storage, but it also limited the formation of additional SEI [166,167]. Therefore, it showed a higher reversible capacity than the single-carbon-coated hollow-shell spheres.

#### 3.2.2. Self-Template Type

By using SiO_2_ as a sacrificial template to prepare a hollow core-shell silicon–carbon composite anode material, acid or alkali was needed as an etching agent, to dissolve the SiO_2_ layer and produce the desired cavity structure. This process was more complicated, and was not only unfavorable regarding environmental concerns but also increased the difficulty of controlling the size of the cavity [158]. During the process of etching SiO_2_ with acid and alkali, some spheres were broken [168]. This adversely affected the subsequent coating and accelerated the collapse of the structure. Therefore, it was necessary to achieve the controllable fabrication of yolk shell structures by removing the template without etching. For example, Si/C [169], HSiNTs/CC [170], SiNPs@C [171], and Si/C [172] had unique hollow structures with sufficient space to alleviate the volume expansion of silicon and facilitate electron-ion transport, enhancing the rate capability and cycle life of LIBs [173,174]. Mi et al. successfully prepared an Si@void@C composite material by using polyethyleneimine (PEI) as a sacrificial template to form the cavity (Figure 6d) [175]. With a coating of single, inorganic nanoparticles to prevent aggregation, the collapse of the cavity was effectively alleviated during the process of pyrolysis, and the cavity was guaranteed, which not only provided a more active site for the storage of lithium ions, but also reduced the diffusion between lithium ions and electrons. As shown in Figure 6e, the surface electron microscope morphology analysis of SiNPs, Si@C, and Si@void@C electrodes after the 5th and 200th cycles showed that the Si@void@C electrode demonstrated the sufficient space for the volume changes. However, since the preparation process of nano-silicon is expensive and complicated and can result in significant losses (>30%) [176,177], it has become necessary to recycle waste silicon [178,179]. Fan et al. prepared a 3D network of Si@C−Ni anode material by recycling silicon waste [75]. As shown in Figure 6f, using a molecular dynamic (MD) simulation of Si@C−Ni, when Li/Si was less than 2.2, the volume change of silicon maintained a linear relationship with Li/Si, which indicated that the structure had not changed. However, when Li/Si was greater than 2.6, cracks appeared in the structure. The result showed that, as compared to Li_4.4_Si (4200 mAh g^−1^), the electrodes made of recycled silicon waste obtained a theoretical capacity greater than 2100 mAh g^−1^ and effectively inhibited the volume expansion of silicon.

With the development of heteroatom-doped carbon materials, research in the field has expanded to include the following: hp−Si@N−C [180], N−P co-doped Si/CNTs/CNFs [181], N−C/Si@G [182], etc. Due to the lower volume expansion of carbon during cycling, the expansion of silicon was suppressed [183,184,185]. The heteroatom doping of carbon materials has included N, F, B, and P [186,187]. Among them, F atoms acted as charge carriers, due to their high electronegativity and strong adhesion [188]. Doping with carbon reduced the resistance of the interface [189]. In addition, the modification of fluorine atoms enabled anions and cations to be absorbed by carbon materials. Fluorine-doped carbon (FC) has shown great potential in the study of anode materials for LIBs [190]. Chen et al. [191] prepared fluorinated core-shell silicon-carbon composites (Si@C) using high-temperature pyrolysis, with PVDF as a dopant. Doping carbon with fluorine and forming Si-F bonds with silicon had a significant effect on suppressing silicon’s volume expansion. Figure 6g shows the surface morphology of n-Si and Si@C electrodes after 50 cycles. Significant cracks appeared on the surface of the n-Si electrode after 50 cycles, while the surface of Si@C electrode was relatively flat, with relatively minor cracks. It further indicated that the formation of the Si-F bond by F doping was beneficial for the mitigation of silicon’s volume expansion. The electrode had an improved long-cycle stability.

**Table 2:** Electrochemical performance of hollow core-shell silicon/carbon anodes for LIBs.

Anode Material	Preparation Method	Current Density^1–2–3^ (mA g^−1^)	Initial Discharge Capacity (mAh g^−1^)	Cycle Number	Cycling Performance/Capacity Retention (mAh g^−1^/%)	Rate Capability (mAh g^−1^)	Refs.
Si@C−Ni		0.1 C–0.1 C–1 C	3317.6	360, 1000	2514.8, 75.8%	1596.9	[75]
Si@void@C	Room temperature solution method	0.1 C–1 C–1 C	2833	1000	1500, 74%	630	[116]
Si@DC	Hydrothermal method	N/A–N/A–100		80	943.8	1260.2	[118]
Si@C@Void@C		100–500–4000	1910	50	1366	1009	[158]
Si@HC	One pot sol-gel method	1000–1000–200	2537.5	2000	500	1060.5	[159]
Si/void/rGO	Modified hummers method	100–200–4000	2381.6	200	1129.2	469.2	[160]
Si@C ⊆ Ni	Solution method	300–300–4800	1382	600	1345	449	[162]
Si/C		100–500–1000	1487	200	650	1197	[169]
HSiNTs/CC	In situ hydrothermal, Hydrolysis method	100–1000–100	1200	100	1026	1489	[170]
SiNPs@C	Colloidal method	N/A–0.1 C–N/A		200	980		[171]
Si/C	Spray drying method, High temperature heat treatment	100–1 C–N/A	936.4	680	80%		[172]
YS Si@mC	Sol–gel method	140–420–8400	1272	400	1000, 78.6%	620	[174]
Si@void@C	Self-sacrifice template strategy	N/A–200–2000		200	854.1	438.8	[175]
Si@C	High temperature pyrolysis	N/A–200–100		50	683, 67%	694.1	[191]

Current density^1–2–3^: ^1^ indicates that the first value is the current density of the first discharge capacity; ^2^ indicates that the second value is the current density of the cycle performance; ^3^ indicates that the third value is the current density of the rate capacity. If there is only one value, this means that the current densities of the three are the same.

The cavity in the hollow core-shell silicon-carbon composite alleviated the volume changes of silicon and effectively protected the carbon layer from cracking, thereby maintaining the integrity of the structure and forming a stable SEI. This type of structure typically has a low density, large surface area, and good chemical durability. Large surface areas improve the rate performance [173,174]. However, the tap density, the volume capacity, and the CE of the structure may decrease. In addition, the preparation of the cavity is usually produced by acid-based etching, which means they are not environmentally friendly [192]. Future research and design in this area will need to be more in-depth.

### 3.3. Porous Structure

The design concept of porous structures is similar to that of a hollow core-shell structure. There were a large number of uniformly distributed cavities between silicon and carbon, which provided sufficient space for the volume expansion of silicon [193,194]. This not only ensured the conservation of the material structure and the rapid attenuation of capacity [195], but also evaded any loss via the agglomeration of silicon particles and interface stress. The electrode material had excellent stability during long cycles [175] (Table 3). In addition, the large surface area and uniformly distributed cavities shortened the diffusion path of lithium ions and enhanced the reactivity and rate capability of the material. Fast charging of the silicon/carbon anode was achieved. Three porous types were determined: (1) porous silicon; (2) porous carbon; and (3) acid-etched porous.

#### 3.3.1. Porous Silicon

**Figure 7:**
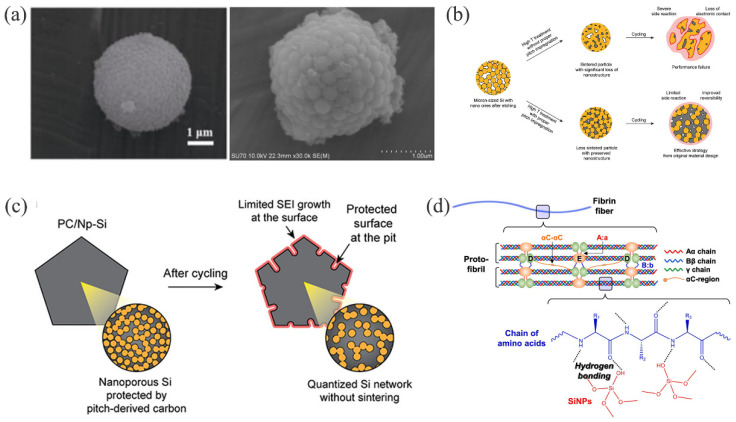
(**a**) SEM images of PSi/C anode materials, before and after deep-cycling [209]. Reprinted with permission from Ref. [209]. Copyright 2021, J. Adv. Ceram. (**b**) Protective mechanism of pitch impregnation on nano-porous silicon structure [74]. Reprinted with permission from Ref. [74]. Copyright 2021, Adv. Mater. (**c**) Structural changes of PC/Np−Si anode before and after cycling [74]. Reprinted with permission from Ref. [74]. Copyright 2021, Adv. Mater. (**d**) Supramolecular interactions between fibrin and silicon [213]. Reprinted with permission from Ref. [213]. Copyright 2021, Appl. Surf. Sci.

Porous silicon [196,197,198] has a three-dimensional pore structure that shortens the lithium ion diffusion path, effectively shortening the battery charging time [199,200] and improving the volume expansion and electrochemical performance [201,202]. The carbon layer coated on the surface of porous silicon buffered the expansion of silicon, while also improving conductivity [203,204,205]. Therefore, porous silicon-carbon composites (e.g., Si/C [206], P−Si@C [207]) have been extensively studied as anodes for high-performance LIBs. Zhang et al. synthesized a hollow structure (PoSi@C−CO_2_) of carbon-coated porous silicon using CO_2_ as the carbon source [208]. The external carbon cage, the middle void space, and the pore of the internal Si adapted to the volume changes of Si and ensured the stability of the external carbon cage, so that the whole composite had significantly less volume expansion during the lithiation process. A pomegranate-type PSi−C [209] composite was prepared by extracting silicon using magnesiothermic reduction. This method controlled the porosity and structure of the silicon [210,211] and produced a small amount of SiC to repair the silicon particles on the C shell, which provided a multi-point contact mode for silicon particles and more Li^+^/e^−^ transmission channels. The formed C−SiC−Si interface had good interface compatibility. The combination of controllable porosity, sufficient void space, and a carbon-shell surface provided ideal conditions for the volume changes of silicon. After analyzing the electrode morphology before and after cycling, as shown in Figure 7a, the structure of the electrode did not significantly change after deep cycling, and the surface roughness was due to the formation of a uniform SEI film during the cycling process. Moreover, the rate test found that when the current density returned to 0.5 A g^−1^, the capacity recovery rate was as high as 100%, which further demonstrated that the presence of sufficient voids inside the PSi/C composite had an excellent buffering effect on the expansion of the silicon electrode.

However, the process of preparing a porous structure is often accompanied by high temperatures, which shrink or even block the resulting voids and decrease the effective utilization of pores. Figure 7b illustrates that Chae et al. successfully synthesized porous Si/C composite (PC/Np−Si) by impregnating pitch-derived carbon into silicon nano-pores, to protect the voids [74]. The required porosity of the composite was reasonably well maintained to accommodate large volume changes, along with a high electrical conductivity to facilitate charge transfer. In a fully lithiated state, the expansion of the electrode was only 60.6%, and the overall structure did not change significantly. In a delithiated state, the thickness of the electrode was similar from the 4th to the 49th cycle, indicating the excellent reversible cycling performance of PC/Np−Si (Figure 7c). Even after 450 cycles, Si and C remained uniformly distributed throughout the particle without pulverization.

#### 3.3.2. Porous Carbon

Porous carbon has enough cavities to accommodate the expansion of silicon particles and prevent the agglomeration that occurs when silicon particles come into contact with each other [203]. The advantages of good conductivity, a large surface area, strong permeability, and stable mechanical properties have attracted much attention. In addition, it can also promote luminescence and electron transport, thereby reducing charge transfer resistance. For example, Si/C/rGO microspheres with a porous structure [195] had a double carbon layer and a porous structure that restricted the volume changes and agglomeration of Si; thus, ensuring the integrity of the electrode structure and improving the cycle stability of the electrode [141,193]. The Si@IHCF [212] composite was obtained through the uniform distribution of silicon on the carbon skeleton, and the porous carbon skeleton could accommodate the volume expansion of SiNPs through its pores. However, as the charge-discharge process was repeated, the SiNPs were shed onto the carbon skeleton, which led to shorter cycle life and faster capacity decay of the electrode. Therefore, Kim et al. successfully developed an Si@C composite material with a three-dimensional porous network structure (3D Si@C) [213] by using the hydrogen bond between the hydroxyl group on silicon and the amide of the fibrin (Figure 7d). Most importantly, this structure had a tunable porosity, which enabled better controllability of the volume expansion of silicon. Fibrin pyrolysis formed a nitrogen-doped carbon with enhanced conductivity [178,214], which was beneficial for lithium-ion storage. As compared to conventional electrodes, the electrochemical performance was significantly improved.

#### 3.3.3. Acid-Etched Porous

Porous composites such as Si@mNOC [215] and Si_40_@C [72] were successfully prepared by acid etching. As anodes of LIBs, the aforementioned composites either stabilize the porous structure of the Si particle by inhibiting the volume expansion and pulverization, or improve the electrochemical property using an appropriate carbon coating. They had excellent reversible capacity, good rate performance, and long cycle life at high current density [215]. Although the electrochemical performance improved using acid-etched porous structures [122,216], the processing method was dangerous and not suitable for large-scale applications. A new Si−C [217] composite material synthesized by sand milling, ball milling, and spray drying did not produce SiO_2_, which eliminated the process of acid-etching. The carbon layer on the surface was a result of the pyrolysis of the polymer, which eliminated contact between the silicon and the electrolyte and promoted the formation of stable SEI [119,122]. For an increased electrochemical performance under high mass loading, Li et al. constructed a rigid-yet-flexible 3D composite structure using Si@SiO_x_/CNTs@C on the surface of Si with CNTs, graphite, and surfactant pyrolytic carbon (SPC) [218]. The volume expansion was suppressed, and the cycle life was extended. Under a current density of 0.42 A g^−1^, there was no capacity loss, as compared to the initial charging capacity after 700 cycles.

**Table 3:** Electrochemical performances of porous silicon/carbon anodes for LIBs.

Anode Material	Preparation Method	Current Density ^1–2–3^ (mA g^−1^)	Initial Discharge Capacity (mAh g^−1^)	Cycle number	Cycling Performance/Capacity retention (mAh g^−1^/%)	Rate Capability (mAh g^−1^)	Refs.
Si_40_@C	Surface modification, Coating, Carbonization, Acid etching	100–1000–100	2629.2	300	994.9, 70.4%	1604.9	[72]
PC/Np−Si	Pitch impregnation method	N/A–0.5 C–N/A		450	80%		[74]
Si/C/rGO	Spray drying	100–500–100	696	70	928	596	[195]
Si/C	Hydrochloric acid etching, Calcination method	100–100–1000	1589.0	50	1287.0	605.9	[206]
P−Si@C	Magnesiothermic, CVD	400–4000–400	2980.2	600	1424	2667	[207]
PoSi@C−CO_2_	CVD	400–400–3200	1301	100	1124, 86.4%	460	[208]
PSi/C	Microemulsion method, Magnesiothermic reduction method	200–1000–200	1313.3	100	629,65%	1400	[209]
Si@IHCFs	Electrospinning	200–200–2000	703	100	903, 89%	743	[212]
3D Si@C	Pyrolysis	100–500–1000	1054	500	54%	730	[213]
Si@mNOC	Sol-gel method	N/A–2000–N/A		4000	900		[215]
Si/C	Sand milling, Ball milling, Spray drying	4.0 mg cm^−2^	471	500	86%	85%	[217]
Si@SiO_x_/CNTs@C	Pyrolysis	420–420–4200	1735.7	700	1740	450.6	[218]

Current density ^1–2–3^: ^1^ indicates that the first value is the current density of the first discharge capacity; ^2^ indicates that the second value is the current density of the cycle performance; ^3^ indicates that the third value is the current density of the rate capacity. If there is only one value, this means that the current densities of the three are the same.

The design of this type of structure has certain similarities to the hollow core-shell structure. The porous structure has abundant cavities, which provide sufficient space for the volume expansion of silicon during the alloying process and relieve the stress caused by the volume expansion. As compared to the hollow core-shell structure, the comparatively smaller surface area and uniformly distributed cavity shorten the diffusion path of Li-ions, improve the rate performance of Si-based electrodes, and have the potential for quick charging-discharging. The tap density and volume capacity are also better and maintain a relatively stable balance. The porous structure can reduce the overall loss of structure caused by the agglomeration due to contact between particles. However, the presence of multiple cavities will form more SEI, resulting in a decrease in the ICE.

### 3.4. Embedded Structures

**Figure 8:**
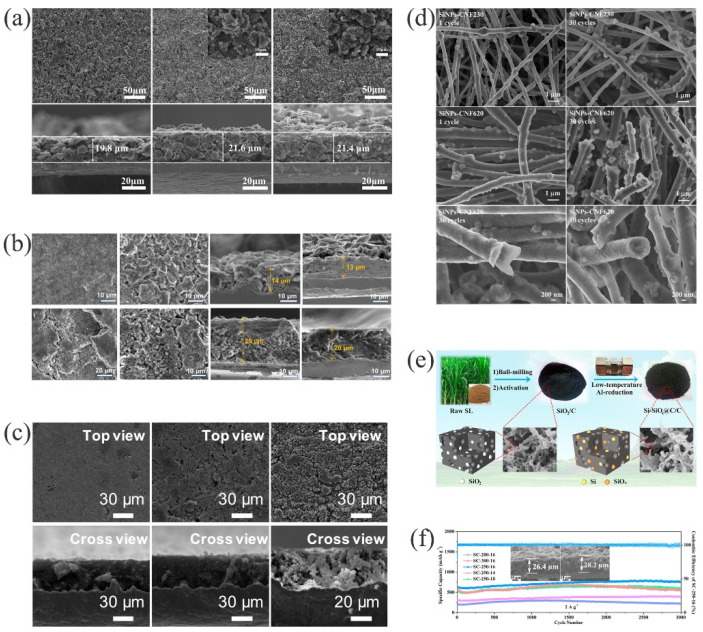
SEM images of (**a**) Si/C electrodes surface before cycle, after 5 and 50 cycles [220]. Reprinted with permission from Ref. [220]. Copyright 2021, J. Alloys Compd. (**b**) pure Si electrode and Si/C electrode before and after 100 cycles [221]. Reprinted with permission from Ref. [221]. Copyright 2020, ACS Appl. Mater. Interfaces. (**c**) SN−MCB electrodes in pristine state, after 100 cycles and after 500 cycles [205]. Reprinted with permission from Ref. [205]. Copyright 2020, Nano Lett. (**d**) SiNPs−CNFs electrodes with different CNF diameters after different cycles [225]. Reprinted with permission from Ref. [225]. Copyright 2020, Electrochim. Acta. (**e**) Preparation process of the Si−SiO_x_@C/C [236]. Reprinted with permission from Ref. [236]. Copyright 2021, Carbon. (**f**) Long cyclic performances [236]. Reprinted with permission from Ref. [236]. Copyright 2021, Carbon.

Embedded silicon-carbon composites have silicon embedded in a continuous carbon matrix [219]. Studies have found that using different carbonaceous substrates as silicon buffer media to adjust for volume changes of silicon during the lithiation/delithiation process and release the mechanical stress can effectively improve the cycle performance of silicon-based anode materials. The voids of the embedded structure can buffer the silicon volume expansion/contraction during the lithiation/delithiation process of lithium ions, and provide a channel for the migration of Li^+^ (Table 4). For example, the volume expansion of Si/C was 9% after 50 cycles [220]. After 100 cycles, the thickness of Si/C electrode increased from 13 μm to 20 μm [221]. And after 500 cycles of SN−MCB (silicon nanoparticles embedded in a micron-sized carbon ball), the electrode thickness increased from 50 μm to 90 μm, while the expansion degree was only 80% [205], as shown in Figure 8a−c. The inlay-derived carbon layer not only provided high electronic conductivity but also significantly reduced the absolute stress/strain, to adapt to the large volume changes and particle agglomeration during repeated lithiation-delithiation [222,223,224].

Ghanooni et al. prepared a self-standing, binder-free, flexible silicon-carbon nanofiber composite (SiNPs−CNFs) by embedding silicon nanoparticles in carbon nanofibers with different fiber diameters via electrospinning [225]. This material effectively avoided the direct exposure of the silicon nanoparticles to the electrolyte and improved the capacity retention during galvanostatic half-cell cycles. As shown in Figure 8d, according to the different diameters of CNFs (230 and 620 nm), the SEM analysis after 1 and 30 cycles showed that the structure of the electrode with a diameter of 230 nm (SiNPs−CNFs230) did not change significantly after 30 cycles, while the structure of the SiNPs−CNFs620 electrode collapsed. The reason for the structural change was that the linear density of the large CNF diameter increased, which caused the agglomeration of the Si NPs, and the larger volume change caused damage to the electrode structure, which had separated the Si NPs from the carbon channels; therefore, the conductivity and capacity of the electrode dropped sharply. To achieve a better application of silicon-based anodes, its volume change should, theoretically, not exceed 70% [226]. For example, the volume change of the SINS/C [227] (~50 nm) structure synthesized by controlling the particle size was approximately 48%. To prevent the rupture of the structure under high-pressure density, the synthesis of a structure with high tap density was studied. Si@C anode materials were synthesized by embedding silicon particles in the matrix of coal tar pitch pyrolytic carbon [228]. The pyrolysis of coal tar pitch effectively reduced the pores and voids in the structure, to limit the volume change. After 250 cycles, the volume expansion rate was 38.6%, with no obvious cracks, while maintaining the structural integrity of the electrode. However, the dispersion of the silicon particles had an effect on the electrical conductivity and mechanical strength of the material. To improve the dispersion of the silicon particles in the carbon matrix, Wang et al. synthesized silico-carbon composites using polyether P123 as a surfactant (P−Si/C) [229]. The introduction of P123 was not only beneficial to the dispersion of silicon in glucose water; the structure also remained unchanged after 30 cycles.

In view of the storage characteristics of renewable natural resources of silicon and considering the economic and environmental concerns involved in industrial production, much research has focused on the development of cheaper biomass for high-value-added silicon-based materials [230,231]. As a traditional food crop, rice is cultivated in most parts of the world, resulting in nearly 100 million tons of rice husk (RH) waste every year [232,233]. Porous network anode materials of Si@SiO_2_@C [234] and Si/C [235] were successfully prepared using RH as the raw material. The stress caused by the volume expansion of silicon was suppressed. However, the preparation of biomass silicon usually involves a high-temperature process, which results in the formation of by-products and structural damage. Chen et al. successfully synthesized Si−SiO_x_@C/C materials using an AlCl_3_ molten salt-assisted, low-temperature aluminothermic reduction method, as shown in Figure 8e [236]. The reversible specific capacity after 3000 cycles at a current density of 2 A g^−1^ was 678.6 mAh g^−1^, with a 6.8% volume expansion (Figure 8f). This study provided a reliable method for the extraction of biomass silicon under low-temperature conditions.

**Table 4:** Electrochemical performance of embedded silicon/carbon anodes for LIBs.

Anode Material	Preparation method	Current Density ^1–2–3^ (mA g^−1^)	Initial Discharge Capacity (mAh g^−1^)	Cycle Number	Cycling Performance/Capacity Retention (mAh g^−1^/%)	Rate Capability (mAh g^−1^)	Refs.
SN−MCB	Scalable microemulsion method	0.05 C–0.2 C–10 C	1800	500	80%	1150	[205]
Si/C	Ball-milling, Annealing	0.2 C	707.8	850	670, 85%	730	[220]
Si/C	Facile coprecipitation method	100–200–100	1016.8	200	584.1	980.8	[221]
SiNPs−CNFs	Electrospinning	N/A–1000–6 C		100	580	242	[225]
SiNS/C	Radio-frequency induction thermal plasma, Spray drying	210–N/A–420	1244 mAh cm^−3^			1029.6 mAh cm^−3^	[227]
Si@C	Ball milling, Liquid solidification method	0.1 C–0.1 C–1 C	1314.6	100	76.70%	1131.9	[228]
P−Si/C	One-step hydrothermal method	500–500–1000	710	100	530	460	[229]
Si@SiO_2_@C	Mechanical milling, Molten salt coactivated magnesiothermic reduction	1000–1000–5000	1044.7	200	973.1, 93.1%	910.2	[234]
Si/C	Aluminothermic reduction	200–500–3000	908	300	460	398	[235]
Si-SiO_x_@C/C	Low-temperature aluminothermic reaction	200	1450.8	400	1562.8	1343.7	[236]

Current density ^1–2–3^: ^1^ indicates that the first value is the current density of the first discharge capacity; ^2^ indicates that the second value is the current density of the cycle performance; ^3^ indicates that the third value is the current density of the rate capacity. If there is only one value, this means that the current densities of the three are the same.

## 4. Conclusions and Perspective

Silicon has a low working potential, abundant reserves, and the highest theoretical specific capacity, and is regarded as the most promising anode material by many researchers. However, due to its significant volume change, it causes active material shedding from the current collector, the pulverization of particles, and the repeated rupturing of the SEI film. As a result, the cycle life and capacity of the electrode are rapidly attenuated, which limits its applications. To address these issues, researchers have considered various solutions, such as adjusting the size and designing porous and hollow structures. The effect of silicon-carbon composites prepared by coupling with carbon materials has proven to be an efficient strategy. In this review, the influences of different designed Si/C anode structures from four perspectives were analyzed. The structure types were coated core-shell structure, hollow core-shell structure, porous structure, and embedded structure. In the design of the structure, the carbon-coated layer, the size of the voids, the control of porosity, and the conductive network formed by chemical bonds promoted adaptations of the volume changes of silicon; thereby ensuring the structural integrity, as well as the formation of stable SEI, and leading to a better electrochemical performance of the anode materials.

Based on researches about Si volume expansion, the following topics were concluded:

(1) Carbon coating can buffer the volume expansion of silicon, but the effect is limited.
(2) The hollow core-shell structure can provide sufficient space for the volume expansion of silicon and has a high-rate performance, due to its large specific surface area; low tap density, volumetric capacity, and CE occurred simultaneously.
(3) A porous structure with abundant cavities can not only provide enough space for the volume expansion of silicon, but it could also reduce the overall loss of the structure caused by agglomeration due to contact between particles. The presence of multiple cavities formed more SEI, resulting in a decrease in the ICE.
(4) The embedded structure can not only buffer the volume expansion of silicon and reduce the agglomeration of silicon, but it can also balance the overall electrochemical performance of the electrode.

Future research should focus on the following aspects: (1) a more uniform distribution of silicon particles; (2) a reasonable design of carbon layer thickness and cavity size; (3) the optimal mass ratio of silicon and carbon; (4) affordability and sustainability; (5) reducing the surface area when designing the silicon volume buffer and conductive network structure; (6) increasing the tap density and compaction density of silicon-carbon materials, to ensure the stability of the electrode structure under high loads; (7) simplifying the preparation methods of silicon-carbon composites so they can be safely produced on a large scale.

Simple and reliable large-capacity silicon-carbon synthesis should be further explored, as Si/C anode materials with more comprehensive properties have significant potential in the development of better, more sustainable lithium batteries.

## Data Availability

Not applicable.

## Abbreviations

LIBs	Lithium-ion batteries
SEI	Solid electrolyte interface
GO	Graphene oxide
RGO	Reduced graphene oxide
XPS	X-ray photoelectron spectroscopy
CVD	Chemical vapor deposition
SEM	Scanning electron microscope
MOFs	Metalorganic frameworks
TEM	Transmission electron microscope
RF	Resorcinol-formaldehyde
PVB	Polyethanol butyral
PEI	Polyethyleneimine
MD	Molecular dynamic
FC	Fluorine-doped carbon
PVDF	Polyvinylidene fluoride
SPC	Surfactant pyrolytic carbon
RH	Rice husk
TMOs	Transition metal oxides
ICE	Initial coulombic efficiency
CE	Coulombic efficiency
CNTs	Carbon nanotubes
CNFs	Carbon nanofibers
SN−MCB	Silicon nanoparticles embedded in a micron-sized carbon ball
